# Intravenous Laser Blood Irradiation Increases Efficacy of Etanercept in Selected Subtypes of Juvenile Idiopathic Arthritis: An Innovative Clinical Research Approach

**DOI:** 10.1155/2013/168134

**Published:** 2013-08-07

**Authors:** Dragos Andrei Chiran, Gerhard Litscher, Michael Weber, Laura Marinela Ailioaie, Constantin Ailioaie, Daniela Litscher

**Affiliations:** ^1^“Grigore T. Popa” University of Medicine and Pharmacy, Faculty of Medicine, 16 Universitatii Street, 700115 Iasi, Romania; ^2^Stronach Research Unit for Complementary and Integrative Laser Medicine, Research Unit of Biomedical Engineering in Anesthesia and Intensive Care Medicine, and TCM Research Center Graz, Medical University Graz, Auenbruggerplatz 29, 8036 Graz, Austria; ^3^Institute for Laser Therapy and Acupuncture, Sohnreystraße 6, 37697 Lauenförde, Germany; ^4^Department of Medical Physics, “Alexandru Ioan Cuza” University, 11 Carol I Boulevard, 700506 Iasi, Romania; ^5^Laser Clinic, 83 Arcu Street, 700135 Iasi, Romania; ^6^St. Mary Emergency Hospital for Children, Second Pediatric Clinic, 62 Vasile Lupu Street, 700309 Iasi, Romania

## Abstract

This single-blind, placebo-controlled study assesses the efficacy of synergic administration of intravenous laser blood irradiation (ILBI) and etanercept in selected subtypes of juvenile idiopathic arthritis (JIA). Etanercept is a tumor necrosis factor alpha blocking agent with recognized importance in JIA. Laser radiation has immunomodulatory effects in animal and human studies. Fourteen patients (Group I) received ILBI and 9 patients (Group II) received placebo laser. ILBI was performed in addition to ongoing JIA medication, including etanercept. ILBI was administrated in 3 sets of 5 consecutive daily sessions, with a 7-week interval between every set of sessions. Evaluation was performed using ACR (American College of Rheumatology) Pediatric Criteria (ACR Pedi) at study enrollment and at 10 and 20 weeks, respectively. After 10 weeks, 85.7% of the patients in Group I fulfilled Pedi 30 criteria, compared to only 55.6% of the patients in Group II. After 20 weeks, all patients in both groups had a Pedi 30 response. In Group I, 92.8% of the subjects met the Pedi 50 response, compared to only 55.6% in the placebo group. One patient in Group I responded best, fulfilling Pedi 70 criteria. If applied synergistically, ILBI and etanercept would have an increased efficacy in promoting JIA remission.

## 1. Introduction

Juvenile idiopathic arthritis (JIA) can be defined as an inflammatory heterogeneous condition, encompassing several subtypes of disease. The term refers to all forms of arthritis which are diagnosed before 16 years of age and last more than 6 weeks [1, 2]. The prevalence of the disease varies a lot regarding the continent, study area, and population, but on average, one in 1000 children worldwide has JIA [3]. 

Even if there is currently no cure for JIA, in the last decade much progress has been made in the therapeutic management, especially with the introduction of biological agents which target specific inflammatory cytokines and signaling molecules [4, 5]. However, Hayward and Wallace reported that only 25% to 40% of patients with JIA achieved inactive disease on biologic medications [4].

**Figure 1:**
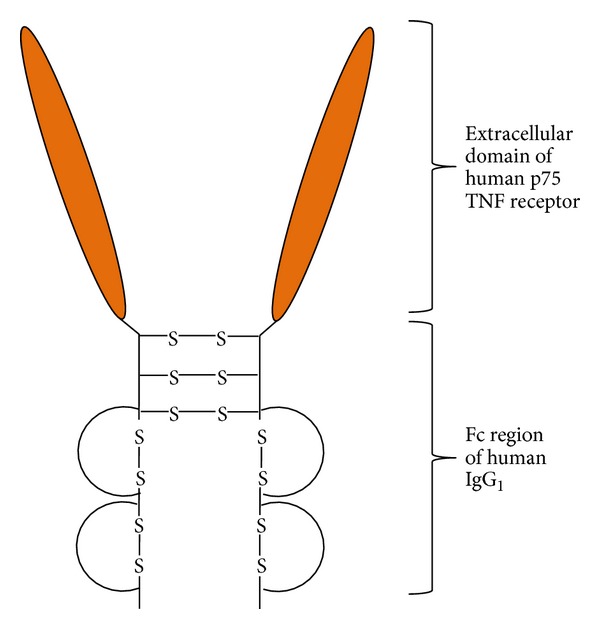
Schematic structure of etanercept. TNF: tumor necrosis factor; Fc: fragment crystallizable; IgG_1_: immunoglobulin G1; S: sulfur.

In this scenario, additional methods which could enhance the efficacy of biological agents appear to be of high importance. It is worth mentioning that biologic medication in general and etanercept, a tumor necrosis factor alpha (TNF*α*) fusion protein inhibitor (see Figure 1) in particular, place a great amount of financial pressure on the health insurance systems, both in Europe and the United States, due to high costs [6, 7].

The importance of etanercept is a fact, being one of the first-line biological drugs to be prescribed in JIA. In 1999, it was the first anti-TNF*α* agent to be approved for use in JIA in the United States [4], and European approval was granted shortly after, in 2000 [8]. 

At the present moment, for example, in Romania, etanercept is the only biologic agent to treat JIA, which has its cost entirely reimbursed by the Romanian National Health Insurance House [9].

It was proved that laser radiation can act on the immune system and decrease serum TNF*α* titers [10]. There is some data regarding the positive effects of intravenous laser blood irradiation (ILBI) in immune-related diseases [11]. There are also some selected case reports and smaller studies of the authors about ILBI in JIA patients, published in proceedings of conferences. 

At present, there is no randomized, placebo-controlled study of this relevance to test the efficacy of synergic administration of ILBI and etanercept in JIA.

## 2. Patients and Methods

### 2.1. Study Setup

This prospective, single-blind, placebo-controlled study was performed during a 19-month period, between November 2011 and May 2013. It was conducted at the Second Pediatric Clinic of the St. Mary Emergency Hospital for Children, Iasi, Romania, with the ethic approval of the aforementioned healthcare institution. Both patients and their families were given complete information; all their questions were answered and all of them signed a written consent.

The eligible patients were 8 to 16 years of age at study enrollment. All of them were diagnosed with JIA using the International League of Associations for Rheumatology (ILAR) criteria [2].

Altogether 23 patients (mean age ± SD: 12.3 ± 2.9 years) were included in this study, presenting moderate and severe forms of JIA, with the following subtypes: extended oligoarthritis, polyarthritis with negative rheumatoid factor (RF−), and polyarthritis with positive rheumatoid factor (RF+). The patients were randomized into two asymmetric groups, using block randomization with an allocation ratio of 3 : 2. Group I (60% of the patients) received ILBI, and Group II (40% of the patients) received placebo laser. This was also combined with stratified randomization to ensure a good balance of arthritis subtypes in each group. The initial demographic data and disease characteristics are shown in Table 1.

The enrolled patients and their parents were fully explained the possible risks and benefits of ILBI. The patients and their parents were fully aware of the existence of the placebo group and they were guaranteed that the ones who would be in the placebo group would be offered, at the end of the study, a real ILBI trial, identical with the one the patients in the laser group had, should they wish.

In this single-blind study, the qualified personnel who performed the laser treatment were aware about the patient adhesion to a specific study group. Therefore, it was of extreme importance for this category of personnel not to influence patients' outcome, and this aspect was a priority for us during the research. The evaluators, who assessed the patients' clinical condition, were not aware of the adhesion of patients to a specific treatment group, in order to avoid any bias.

The exclusion criteria were formed of three main groups. Exclusion criteria due to selected subtypes of JIA included in the study: forms of systemic arthritis, enthesitis-related arthritis, persistent oligoarthritis, psoriatic arthritis, undifferentiated arthritis, and positive diagnosis of uveitis [2]. The second group of exclusion criteria was due to contraindications and special warnings to etanercept therapy: pregnant or sexually active female patients without using effective contraception (this situation is considered to be rare below 16 years of age, but still, it should be given proper attention), active infections, risk of sepsis, history of tuberculosis and hepatitis, with B or C virus, malignancies, and lymphoproliferative disorders. These conditions represent key points, when etanercept is to be initiated, and special medical conduct is performed if one of the above occurs, according to the national guidelines and manufacturer's advice for Europe [8, 9]. The last group of exclusion criteria was related to ILBI and consisted of sensitivity to light, history of epilepsy, and age under 8 years. The last requirement was implemented due to the fact that little children have low compliance to peripheral intravenous (i.v.) line insertion and have very limited understanding of this study setup, even if their parents would have fully agreed with all the research protocols.

The main inclusion criterion was ongoing anti-TNF*α* therapy with etanercept for all the patients for at least 3 months, without obtaining an improvement of at least ACR (American College of Rheumatology) Pediatric 30 response [12], during the last 3 months. The other inclusion criteria were active arthritis for at least 6 months, the absence of remission in the last 6 months, as defined by Wallace et al. [13], and age range between 8 and 16 years at the enrollment in the study.

Analgesics, including opiates, anti-inflammatory medication (steroidal and nonsteroidal), and disease-modifying anti-rheumatic drugs (DMARDs) were allowed. Detailed information is displayed in Table 2.

All patients were enrolled in a physical therapy program and psychological support was available throughout the whole period of the study.

### 2.2. Laser Equipment and Protocol

Both subjects in Group I—ILBI (14 patients) and in Group II—placebo (9 patients) received the same standard i.v. laser protocol, up to the very moment when the light radiation was delivered through optical fiber into the vein. The placebo patients did not effectively receive the laser radiation through the already connected intravenous optical fiber.

Patients did not know if they were receiving laser radiation or placebo, and confidentiality was assured with laser protective goggles worn by the patients.

Laser therapy was given in 3 sets of 5 consecutive daily sessions, with a 7-week interval between every set of sessions.

**Figure 2:**
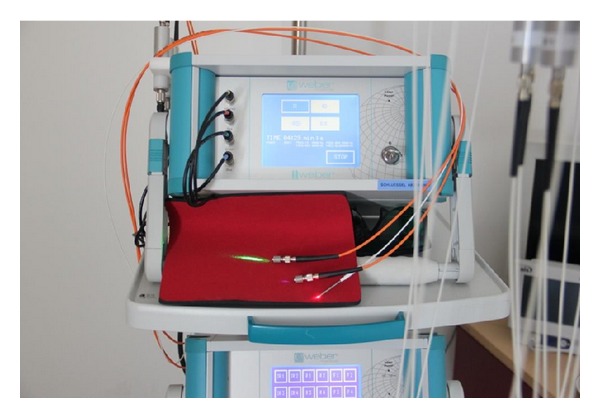
Weberneedle Endolaser (identical systems are available at the Medical University of Graz and the St. Mary Emergency Hospital for Children Iasi). The following wavelengths were used: 630 nm (red), 536 nm (green), and 405 nm (violet).

ILBI protocol consisted of 3 different wavelengths (630 nm, 536 nm, and 405 nm) with a 5 mW maximum output power in continuous mode (Figure 2). Radiation was given for 10 minutes for each wavelength, with a total duration of 30 minutes per session. The three types of radiation, regarding the wavelength, were given in the following order: red radiation (630 nm) at the beginning of the session, followed by green radiation (536 nm) and violet radiation (405 nm) at the end. This protocol was implemented for all patients and for all laser sessions performed in the study. The reason for this choice of protocol was to deliver an increasing amount of energy to the blood stream, as photons with shorter wavelengths carry a greater amount of energy compared to photons with longer wavelength. All patients were lying comfortably on a treatment bed during therapy.

The site for the i.v. access was preferred at the cubital region, but if no vein could be located, alternate sites at the forearm or the dorsal region of the hand were used. Large veins at palpation were the standard of choice.

**Figure 3:**
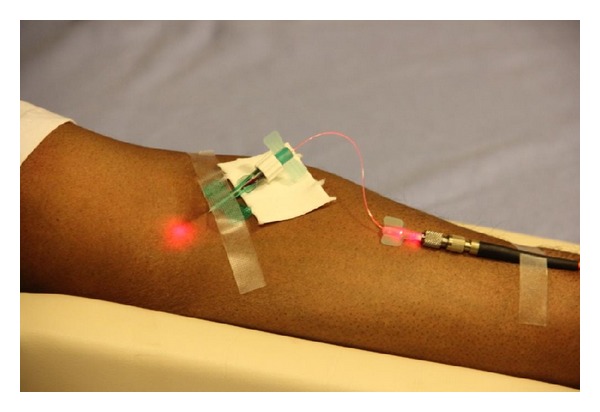
Application of the Weberneedle Endolaser on a patient.

The laser radiation was delivered with a sterile optical fiber i.v. catheter, which was passing through the lumen of a butterfly needle (size 21G) into a peripheral vein. The butterfly needle and the external tip of the optical fiber catheter were immobilized to the patient's skin with adhesive tape (Figure 3).

Prior to procedure, the needle insertion site was cleansed with disinfectant solution. Approximately 2 g of topical anesthetic cream (Lidocaine 2.5%/Prilocaine 2.5%) was applied on that site to prevent the pain caused by the needle. Then, a sterile transparent film dressing was applied on the top to allow the anesthetic to work effectively, before obtaining venous access. When the ILBI procedure was finished, the butterfly and the optical fiber catheter were extracted, and an adhesive sterile dressing was applied on the site.

### 2.3. Assessment and Statistical Analysis

Patients were assessed initially, when enrolled in the study. Two more assessments were performed at 10 weeks and 20 weeks, respectively, from the initial time. Following the ILBI treatment schedule, it results that the second assessment was done one week after the second set of sessions, and the last evaluation was at 3-week time after the last set of sessions.

All the patients in the placebo group wanted to get benefit of verum ILBI therapy at the end of the study, and we managed to record data for five of those patients, and four patients are still undergoing the ILBI protocol at the present moment. These findings are not the objective of the present study and they will not be presented in this paper.

Each clinical assessment included the six variables of the ACR core set data for JIA [12]: the number of joints with active arthritis, the number of joints with limited range of motion, the physician's global assessment of disease activity on a visual analogue scale (VAS), the parent's or patient's global assessment of overall well-being on a VAS, physical function (assessed with the Childhood Health Assessment Questionnaire (CHAQ) [14], with scores ranging from 0 to 3 for 8 activities of daily living), and the erythrocyte sedimentation rate (ESR). The original VAS proposed by Giannini et al. [12] had a range from 0 to 100 mm, with 0 being the best score. We kept the length of the scale, but we modified its basic length unit from millimeters to centimeters, obtaining a scale ranging from 0 to 10 cm. We found this rescaling very useful, especially when the child is pointing with his finger for scoring. Not only would it have been laborious to identify the exact millimeter a child is pointing at, but it is also difficult to ask a child to adhere to a scale with so many scaling units. Approximation was performed to the closest unit in centimeters, when the patient finger was placed between two consecutive units. To maintain the uniformity, the VAS for the physician assessment was modified in the same way, and instructions for approximation were given accordingly.

Disease improvement was evaluated using the ACR Pediatric (ACR Pedi) criteria. The ACR Pedi 30 (50, 70, and 90, resp.) criteria are defined as improvement of more than 30% (50%, 70%, and 90%, resp.), in at least 3 of the 6 core set variables used to assess disease activity, with no more than one variable worsening by more than 30% [12].

The median values and the standard deviation were calculated for all quantifiable data in both groups. The differences among each group regarding the initial parameters were evaluated using a 2-sample homoscedastic *t*-test for a confidence interval of 95%. The disease outcome for each group was tested for significant differences using a 2-sample heteroscedastic *t*-test. A value of *P* < 0.05 was considered significant, and a value of *P* ≥ 0.05 was considered non-significant. The basic statistical analysis and chart generation were done using MS Excel 2010 software.

## 3. Results

The initial demographic data and disease characteristics were balanced among the two groups, with no statistically significant differences (see Table 1).

After 10 weeks, the patients treated with ILBI displayed a better improvement in all six parameters of the ACR core set data compared to the placebo group (see Table 3).

**Figure 4:**
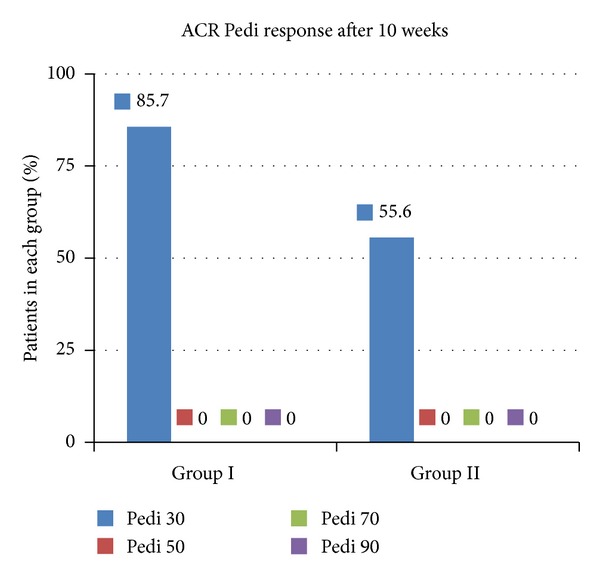
ACR Pedi response at the evaluation performed at 10 weeks from ILBI initiation.

Regarding the ACR Pedi criteria, 85.7% of the patients in Group I, compared to only 55.6% of the patients in Group II, managed to meet the Pedi 30 response (see Figure 4). None of the patients in both groups met the Pedi 50, 70, or 90 responses.

At the final evaluation, the ILBI patients continued to display a more significant improvement in comparison with the patients in the control group in all aspects encompassed in the ACR core set data (see Table 4). 

**Figure 5:**
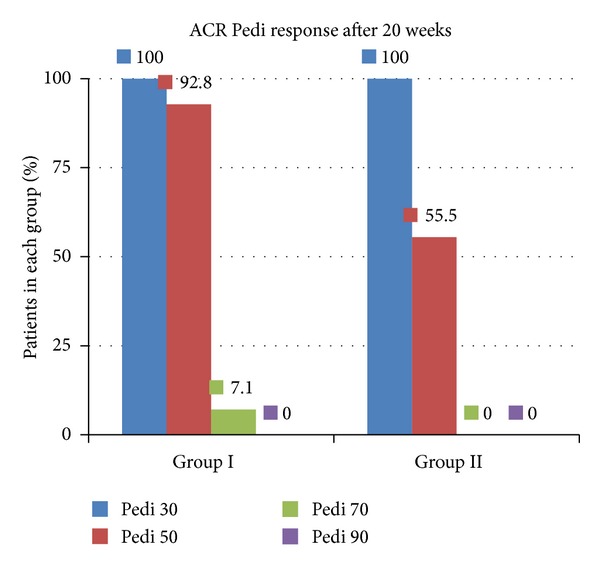
ACR Pedi response at the evaluation performed at 20 weeks from ILBI initiation.

All patients from both groups fulfilled the Pedi 30 response. In Group I, 92.8% of the subjects met the Pedi 50 response, compared to only 55.6% in the placebo group. Only one patient in Group I had a Pedi 70 response, and none of the patients from the present research had a Pedi 90 response (see Figure 5) at the end of the study.

## 4. Discussion

JIA is still a pathological condition with no clear etiology, and a multifactorial approach is preferred to explain the causes of the disease [1]. The treatment evolved over time from nonsteroidal anti-inflammatory drugs (NSAIDs) and corticosteroids to classic DMARDs like Methotrexate and Sulfasalazine and to biological agents in the last 15 years. Biological agents target specific inflammatory cytokines involved in JIA, like TNF*α*, interleukin 1 (IL1), and IL6, as well as signaling molecules involved in the regulation of B-cell and T-cell lymphocyte responses [4, 5].

The main disadvantage of biological agents is their immunosuppressant effect which leads to an increased risk of opportunistic infections, from mild to severe (tuberculosis, viral hepatitis reactivation), and rarely to malignancies. However, long-term safety and effectiveness of etanercept in JIA are widely recognized [4, 5, 15]. 

There have been some debates over the doses of etanercept in JIA. Increased doses of etanercept may not offer any additional benefit in children with unsatisfactory response to the standard dose [16].

 Due to administrative or financial reasons, it may not be possible to change the biologic agent in such cases [9], so finding a synergistic therapeutic method to increase etanercept efficacy represents a major challenge. Our results proved that ILBI had indeed a beneficial effect for the patients in Group I, who displayed a better disease improvement throughout the study. At baseline, there were no significant differences between the two groups in terms of statistical analysis. The much better outcome in the ILBI group was statistically significant compared to the results obtained in the placebo group, while all patients continuously received etanercept.

In our study design, we excluded some subtypes of JIA. We focused on the polyarticular forms of the disease, due to their more severe evolution, excluding forms of persistent oligoarthritis. Psoriatic arthritis and enthesitis-related arthritis each represent only 1–11% of the JIA patients [1, 2]. IL6 receptor inhibition is considered more effective in systemic arthritis [17]. Patients with uveitis were excluded, as there are opinions more favorable to Infliximab [18] and Adalimumab [19] (other anti-TNF*α* agents), or Abatacept [20] (a cytotoxic T-lymphocyte antigen 4 fusion protein), in the treatment of JIA-associated uveitis.

Regarding steroidal medication, all patients from both groups received at least a form of corticosteroid (oral, i.v. pulsed, intraarticular) when enrolled in the study. The local practice protocol was to decrease the dose or limit the use of steroidal medication due to the associated high toxicity. When a Pedi 30 response was achieved, tapering of the steroid dose or limiting it to intraarticular administration was performed. A Pedi 50 or Pedi 70 response was a clear indication for stopping the corticosteroid medication with gradual withdrawing. Since steroid medication management and Pedi response are so closely related to one another, ILBI patients managed to reduce and avoid further corticosteroids in a much larger percent compared to placebo patients (see Figures 4 and 5).

The placebo group (after 10 or 20 weeks, resp.) achieved a disease improvement rate comparable to the data in the literature [21]. The small differences in outcome could be explained by the size of the placebo group and by the fact that not all the patients had the same length of etanercept administration period at enrolment in the study.

At both evaluations, after 10 weeks and at the end of the study, ILBI patients displayed a better improvement in all the ACR core set parameters. All the differences were statistically significant (*P* < 0.05), with the exception of the score for physician's global assessment of disease activity after 10 weeks, where *P* had a value of 0.268. 

All patients from both groups fulfilled the Pedi 30 response at the end of the study, which certifies the value of etanercept administration. The result of 92.8% of the subjects who met the Pedi 50 response after synergistic ILBI, compared to only 55.6% patients in the placebo group, proved the importance of this new therapeutic approach in JIA. Moreover, only one patient in the ILBI group had a Pedi 70 response, whereas no patient with placebo laser obtained this outcome. None of the patients from the present research had a Pedi 90 response (see Figure 5).

No side effects to be correlated with ILBI were observed. Possible initial needle fear was overcome with thorough skin desensitization, using a topical anesthetic cream. Minor upper respiratory infections and skin reactions at the etanercept subcutaneous injection site were observed in 3 patients in Group I and in 2 patients in Group II. With appropriate treatment (antibiotics and antihistamines, resp.), all patients could continue the study without interrupting etanercept or ILBI. These were common side effects to etanercept [4, 5, 15, 21].

Trying to explain how ILBI could promote a better outcome when given together with etanercept leads to the molecule targeted by this biologic agent. TNF*α* is a powerful proinflammatory cytokine and has increased titers, both in serum and in the synovial fluid of JIA patients. It exists in a soluble (sTNF*α*) and a membrane-attached form (mTNF*α*). TNF-mediated biology gains additional complexity from the distinct signaling pathways, mediated through two types of receptors, TNFR1 and TNFR2, which can also exist in circulating and membrane-attached forms. Both receptors bind all TNF*α* molecules plus soluble lymphotoxin alpha 3 (LT*α*3) and cell-surface LT*α*2*β*1, as LT, formerly called TNF*β*, is structurally similar to TNF*α*. Moreover, mTNF*α* can also function as a receptor when a circulating endogenous TNF*α* receptor or an appropriate biological agent binds to it [22, 23].

Etanercept (Enbrel, as trade name) is a fusion protein consisting of the extracellular domain of the TNFR2 combined with the Fc portion of the human immunoglobulin IgG1. Etanercept binds to sTNF*α* and mTNF*α* and thus decreases the inflammatory TNF*α*-mediated signaling [4]. Because of its structure, it also binds to LT*α*3 and LT*α*2*β*1 [22].

Intravenous laser irradiation was reported to change physiological parameters in a rat model [24]. Laser radiation was also demonstrated to reduce sTNF*α* in some animal studies with experimentally induced acute inflammation in lungs [10, 25]. Mesquita-Ferrari et al. illustrated that low-level laser therapy (LLLT) caused a decrease in TNF*α* mRNA (messenger ribonucleic acid) expression at 1 and 7 days following the cryoinjury of tibialis anterior muscle in rats [26]. Decreasing TNF*α* mRNA expression in the affected muscular cells also decreases both sTNF*α* and mTNF*α*, as sTNF*α* results from enzymatical cleavage of mTNF*α* [22]. LLLT also proved to decrease the mRNA level of mTNF*α* in *in vitro* synoviocytes from rheumatoid arthritis patients [27]. 

According to these experiments, ILBI would be capable of decreasing sTNF*α* titers and mTNF*α* membrane presentation. Thus, we assume that the constant quantity of etanercept, therapeutically given to the patient, would be able to inactivate most of the TNF*α* molecules with a higher probability.

A pretty new research field is represented by the role played by LT family in inflammation in general and in arthritis in particular [28]. Etanercept neutralizes LT*α*3 and sTNF*α* with similar potency, and so, neutralization of sTNF*α* could be reduced by competition, if concentrations of LT*α*3 are high. There is no evidence to date that LT blockade provides etanercept with any therapeutic advantage [22].

Searching the present scientific literature, we discovered that to the best of our knowledge no one studied the interaction between laser radiation and LT. Taking into account that laser therapy is reducing the serum titers of the major pro-inflammatory cytokines [10, 25–27], it is worth raising the question if ILBI could modulate the immune response by also decreasing the LT*α*3 and LT*α*2*β*1 production.

In such an instance, ILBI could enhance etanercept efficacy by decreasing the competition between TNF*α* and the LT family, leading to a better overall patient response.

An emerging area of interest, regarding etanercept mechanisms of action, centers on the functional outcomes of its interaction with mTNF*α*. Current evidence suggests that etanercept acts both as an antagonist, by blocking mTNF*α* interaction with TNFR1 or TNFR2, and as an agonist, by initiating reverse signaling. The latter type of signaling leads to apoptosis, cytokine suppression, or cell activation [23]. 

Decoster et al. demonstrated that TNF*α* is firstly formed as a membrane-bound protein, which is responsible for receptor downmodulation [29]. Laser radiation could promote receptor down-modulation, in the case of cellular activation, due to the binding of etanercept to mTNF*α* [23]. This would result in a smaller probability of occurrence for further TNF*α*-induced reactions in those cells.

Another pathway of action for ILBI, in balancing the inflammation, is by increasing anti-inflammatory cytokines titers [30]. A recent animal *in vivo* study observed statistically significant beneficial anti-inflammatory effects of LLLT administration in induced rheumatoid arthritis in rats [31].

It is likely that several of the above mechanisms act in concert. The contribution of ILBI to the various etanercept acting mechanisms, coupled to its balancing action on enhancing the anti-inflammatory cytokines pathways, remains a focus subject for the scientific community.

ILBI, firstly discovered by Russian scientists in 1981, still represents a novel treatment modality amongst the applications of lasers in medicine. This is because the studies were published mainly in Russian and remained mostly unknown to the Western Europe and United States [11]. Within the last 10 years, ILBI started to prove its efficacy in a wide range of medical conditions like diabetes mellitus, chronic hepatitis, hepatic cirrhosis, dyslipidemia, cardiovascular diseases, and autoimmune diseases [11].

Schumm indicates that ILBI can successfully be applied in multiple sclerosis, leading to a highly significant improvement in the quality of life of the treated patients [32]. Last year, Huang et al. reported the beneficial effects of ILBI therapy on oxidative stress and mitochondrial dysfunction in subjects with chronic spinal cord injury resulting from trauma [33].

ILBI was also reported to be a valuable adjuvant in oncology due to its immunomodulatory effects [11]. Relatively new data places this therapeutic modality in the field of sports medicine, promoting significant improvement in the sleep pattern, vigilance, and overall physical performance of athletes [34].

A new challenge is represented by the transcutaneous and transmucosal (sublingual) laser blood irradiation due to the noninvasive aspect of the therapy. Under certain conditions, these noninvasive methods can have similar efficacy compared to ILBI, and it can be the only alternative to incompliant patients to vein puncture [35]. 

## 5. Conclusions

ILBI and etanercept have an increased efficacy in promoting the remission of selected subtypes of JIA, if applied synergistically. Our significant results proved the value of ILBI in cases of moderate-to-severe polyarthritis.

Further studies regarding laser therapy interaction with TNF*α* and the LT cytokine family might explain its anti-inflammatory effect more accurately.

## Figures and Tables

**Table 1:** Initial demographic data and disease characteristics.

Characteristic	Group I—ILBI (*n* = 14)	Group II—Placebo (*n* = 9)	Statistical significance
Demographic			
Male sex—no. (%)	8 (57.1)	5 (55.6)	NS
Age—years; mean ± SD	12.1 ± 3.2	12.5 ± 2.6	NS
Weight—kg; mean ± SD	30.0 ± 14.8	33.6 ± 18.6	NS
JIA characteristics			
Duration of disease—years; mean ± SD	4.1 ± 2.4	4.2 ± 3.0	NS
Extended oligoarthritis—no. (%)	6 (42.9)	4 (44.4)	NS
Polyarthritis (RF negative)—no. (%)	5 (35.7)	3 (33.3)	NS
Polyarthritis (RF positive)—no. (%)	3 (21.4)	2 (22.2)	NS

NS: nonsignificant difference between the two groups, *P* value ≥ 0.05. SD: standard deviation. RF: rheumatoid factor.

**Table 2:** Arthritis-related pharmacological therapy.

Characteristic	Group I—ILBI (*n* = 14)	Group II—Placebo (*n* = 9)	Statistical confidence
*Current administration of DMARDs *			
Methotrexate—no. (%)	12 (85.6)	7 (77.8)	NS
Methotrexate mean dose ± SD per patient—mg/m^2^/week	13.7 ± 5.2	14.2 ± 4.9	NS
*All patients receiving Methotrexate were also prescribed folic acid 5 mg, which was given the morning following Methotrexate administration, as per local protocol *			
Other DMARDs—no. (%)	0 (0)	0 (0)	NS
*Current administration of corticosteroids *			
Oral corticosteroid—no. (%)	10 (71.4)	7 (77.8)	NS
Oral corticosteroid—Prednisolone equivalent mean dose ± SD per patient—mg/kg/day	0.28 ± 0.15	0.28 ± 0.18	NS
I.v. Prednisolone—no. (%)	4 (28.6)	2 (22.2)	NS
I.v. Prednisolone—no. of boluses per month per patient (30 mg/kg, max 1 g); mean ± SD	3.2 ± 1.2	3.4 ± 1.3	NS
Intraarticular Prednisolone—no. (%)	3 (21.4)	3 (33.3)	NS
Intraarticular Prednisolone—no. of joints per patient (1.5–2.5 mg/small joint; 25–50 mg/large joints); mean ± SD	5.1 ± 2.3	4.6 ± 1.9	NS
*Current administration of biological agents *			
Etanercept—no. (%)	14 (100)	9 (100)	NS
Etanercept mean dose ± SD per patient—mg/kg, twice a week	0.4 ± 0.0	0.4 ± 0.0	NS
Other biological agents—no. (%)	0	0	NS

NS: nonsignificant difference between the two groups, *P* value ≥ 0.05. DMARDs: disease-modifying antirheumatic drugs.

**Table 3:** Evolution of ACR core set of variables at 10 weeks from initiation of ILBI.

Variable	Group I—ILBI (*n* = 14)		Group II—Placebo (*n* = 9)		Statistical comparison among groups	
	Initial	After 10 weeks	Initial	After 10 weeks	Initial	After 10 weeks
No. of joints with active arthritis	9.9 ± 3.5	6.4 ± 2.3	10.1 ± 2.1	8.2 ± 2.4	*P* = 0.890	*P* = 0.044
No. of joints with limited range of motion	38.5 ± 5.6	29.9 ± 5.4	38.7 ± 4.8	34.6 ± 4.6	*P* = 0.942	*P* = 0.021
Score for physician's global assessment of disease activity	8.4 ± 1.2	6.1 ± 1.8	8.7 ± 0.9	6.7 ± 1.0	*P* = 0.498	*P* = 0.268
Score for parent's or patient's global assessment of overall wellbeing	8.6 ± 1.0	5.7 ± 0.8	9.1 ± 0.9	6.3 ± 0.9	*P* = 0.213	*P* = 0.049
CHAQ score	14.9 ± 2.6	9.4 ± 2.3	14.0 ± 2.2	11.3 ± 2.7	*P* = 0.390	*P* = 0.041
ESR—mm/hr	50.6 ± 24.8	30.9 ± 11.4	51.6 ± 15.6	38.8 ± 7.8	*P* = 0.922	*P* = 0.042

CHAQ: Childhood Health Assessment Questionnaire. ESR: erythrocyte sedimentation rate.

**Table 4:** Evolution of ACR core set of variables at 20 weeks from initiation of ILBI.

Variable	Group I—ILBI (*n* = 14)		Group II—Placebo (*n* = 9)		Statistic comparison among groups	
	Initial	After 20 weeks	Initial	After 20 weeks	Initial	After 20 weeks
No. of joints with active arthritis	9.9 ± 3.5	4.4 ± 1.5	10.1 ± 2.1	5.8 ± 2.0	*P* = 0.890	*P* = 0.034
No. of joints with limited range of motion	38.5 ± 5.6	16.3 ± 4.7	38.71 ± 4.8	24.3 ± 7.7	*P* = 0.942	*P* = 0.002
Score for physician's global assessment of disease activity	8.4 ± 1.2	3.1 ± 0.9	8.7 ± 0.9	3.9 ± 1.1	*P* = 0.498	*P* = 0.046
Score for parent's or patient's global assessment of overall wellbeing	8.6 ± 1.0	3.4 ± 1.0	9.1 ± 0.9	4.2 ± 1.3	*P* = 0.213	*P* = 0.043
CHAQ score	14.9 ± 2.6	5.7 ± 1.1	14.0 ± 2.2	6.9 ± 1.3	*P* = 0.390	*P* = 0.013
ESR—mm/hr	50.6 ± 24.8	15.9 ± 7.9	51.6 ± 15.6	22.9 ± 11.1	*P* = 0.922	*P* = 0.045

CHAQ: Childhood Health Assessment Questionnaire. ESR: erythrocyte sedimentation rate.
